# Incidence and Risk Factors of the COVID-19 Pandemic: An Epidemiological Approach

**DOI:** 10.3390/epidemiologia4020014

**Published:** 2023-04-25

**Authors:** Alberto Arnedo-Pena, Francisco Guillen-Grima

**Affiliations:** 1 Epidemiology Division, Public Health Center, 12003 Castelló de la Plana, Spain; 2 Public Health and Epidemiology (CIBERESP), 28029 Madrid, Spain; 3 Department of Health Sciences, Public University of Navarra, 31006 Pamplona, Spain

After three years of the COVID-19 pandemic, it is certain that the SARS-CoV-2 virus has been a turning point for humanity in both developed and developing countries. The worldwide reports of mortality and incidence figures related to the COVID-19 pandemic, with mortality rates of 0.85–0.86 per 10^3^ inhabitants, attack rates of 84.2–94.6 per 10^3^ inhabitants, and fatality–case rates of 0.90–1.0%, may only weakly reflect the real figures [1,2]. In 2021, a study estimated that more than 40% of the world’s population has been infected by the virus [3]. Epidemiological models such as those used by Our World in Data [4], by using various data sources and assumptions, provide mean estimates of the true number of infections. In addition, the pandemic has caused substantial damage worldwide, with significant differences in the level of damage between countries and within countries regarding COVID-19 incidence and death rates, vaccinations, and non-pharmacological measures against the pandemic, with low-income countries and vulnerable groups being the most heavily affected [5,6].

The epidemiology of COVID-19 was the scope of a Special Issue in *Epidemiologia*, entitled “Genetic, Lifestyle, Socio-Economic, and Environmental Risk Factors Associated with the Incidence of the COVID-19 Pandemic”. Four of the eight published manuscripts focused on high-risk groups in areas of society most affected by the pandemic: staff and residents in residential long-term care homes (LTCHs) for older people and patients with chronic mental illness [7,8,9,10]. The remaining manuscripts focused on the secondary rate of COVID-19 transmission in the families of healthcare workers [11], ABO blood groups as a potential risk factor for infection [12], post-COVID-19 complications [13], and new SARS-CoV-2 variants [14]. All of these studies were conducted before the vaccination program against SARS-CoV-2 began in January 2021.

Three articles described the characteristics of residents, staffing, and facilities in LTCHs during the COVID-19 pandemic in three areas of Spain: Madrid, Catalonia, and Castellon. In Castellon, the cumulative incidence rate (CIR) was 34.8% in residents and 19.2% in staff [9]; the COVID-19 mortality rate was 8.7 and 9.2% in Catalonia [8]; and 18.3% of the total mortality occurred in Madrid [7]. For patients with chronic mental illness, the CIR was 21.7%, and for staff, it was 15.4% [10]. The occupancy rate, LTCH size, private and public–private partnership, and COVID-19 CIR in counties where LTCHs were located were factors associated with mortality in three studies. The infection of staff with COVID-19, high ratios of residents/staff, the age of the facilities, the crowding index, private ownership, and severe disability in residents were associated with the incidence of COVID-19 [9,10].

Many factors associated with COVID-19 incidence and mortality can be prevented, for example, by increasing infection and quality control, improving residents/staff ratios and structural facilities with an optimal size, and augmenting the public ownership of LTCHs. An optimal LTCH size was recommended to be between 30 and 70 places [8]. Vaccines against SARS-CoV-2 have been essential in reducing the severity of COVID-19 infection and the number of related deaths in LTCHs. However, the transmission of the virus has continued with high intensity in these places [15,16], and reinfection could increase the risk of complications in these populations [17].

Healthcare workers were found to be another group at high risk of COVID-19, with a secondary transmission rate of 27.3% in their household [11]; when healthcare workers had an individual room, the transmission risk decreased. ABO blood groups and SARS-CoV-2 incidence were studied during mass gathering events during the Falles festival of Borriana (Spain), but significant associations were not found [12]. Post-COVID-19 complications in these participants were studied during a 6-month follow-up; 33.1% experienced at least one complication, and 29.4% of the participants sought medical assistance [13]. Finally, potential new COVID-19 variants could change our approach to the virus, and current strategies must address these situations [14]. Efforts to prevent and control COVID-19 and potential future pandemics should continue in all countries, with special attention being paid to high-risk groups such as residents of LTCHs and healthcare workers. Finally, highlighting the need for research, surveillance, and monitoring of new variants and emerging diseases will emphasize the ongoing importance of the field of epidemiology in safeguarding global health.

## References

[1] Center for Systems Science and Engineering (CSSE) at Johns Hopkins University (JHU) COVID-19 Dashboard. https://coronavirus.jhu.edu/map.html.

[2] World Health Organization WHO Coronavirus Disease (COVID-19) Dashboard. https://covid19.who.int/.

[3] COVID-19 Cumulative Infection Collaborators (2022). Estimating global, regional, and national daily and cumulative infections with SARS-CoV-2 through Nov 14, 2021: A statistical analysis. Lancet.

[4] Our World in Data. Dashboard. https://ourworldindata.org/grapher/daily-new-estimated-infections-of-covid-19.

[5] Aung M.N., Koyanagi Y., Yuasa M. (2021). Health inequality among different economies during early phase of COVID-19 pandemic. J. Egypt. Public Health Assoc..

[6] Dang H.H., Malesky E., Nguyen C.V. (2022). Inequality and support for government responses to COVID-19. PLoS ONE.

[7] Zunzunegui M.V., Rico M., Béland F., García-López F.J. (2022). The impact of long-term care home ownership and administration type on all-cause mortality from March to April 2020 in Madrid, Spain. Epidemiologia.

[8] Zunzunegui M.V., Béland F., Rico M., Garcia-López F.J. (2022). Long-term care home size association with COVID-19 infection and mortality in Catalonia in March and April 2020. Epidemiologia.

[9] Arnedo-Pena A., Romeu-Garcia M.A., Gascó-Laborda J.C., Meseguer-Ferrer N., Safont-Adsuara L., Prades-Vila L., Flores-Medina M., Rusen V., Tirado-Balaguer M.D., Sabater-Vidal S. (2022). Incidence, mortality, and risk factors of COVID-19 in nursing homes. Epidemiologia.

[10] Arnedo-Pena A., Romeu-Garcia M.A., Gasco-Laborda J.C., Meseguer-Ferrer N., Safont-Adsuara L., Guillen-Grima F., Tirado-Balaguer M.D., Sabater-Vidal S., Gil-Fortuño M., Pérez-Olaso O. (2022). Incidence, hospitalization, mortality and risk factors of COVID-19 in long-term care residential homes for patients with chronic mental illness. Epidemiologia.

[11] Remón-Berrade M., Guillen-Aguinaga S., Sarrate-Adot I., Garcia-Garcia M.P., Lerga-Berruezo M.d.C., Guillen-Aguinaga L., Guillen-Grima F. (2022). Risk of secondary household transmission of COVID-19 from health care workers in a hospital in Spain. Epidemiologia.

[12] Domènech-Montoliu S., Puig-Barberà J., Pac-Sa M.R., Vidal-Utrillas P., Latorre-Poveda M., Del Rio-González A., Ferrando-Rubert S., Ferrer-Abad G., Sánchez-Urbano M., Aparisi-Esteve L. (2022). Complications post-COVID-19 and risk factors among patients after six months of a SARS-CoV-2 infection: A population-based prospective cohort study. Epidemiologia.

[13] Domènech-Montoliu S., Puig-Barberà J., Guerra-Murcia O., Pac-Sa M.R., Orrico-Sanchéz A., Gómez-Lanas L., Sala-Trull D., Domènech-Leon C., Del Rio-González A., Sánchez-Urbano M. (2023). ABO blood groups and incidence of COVID-19 in the mass gathering events in Borriana (Spain), March 2020: A retrospective cohort study. Epidemiologia.

[14] Redwan E.M., Elrashdy F., Aljabali A.A.A., Baetas-da-Cruz W., Barh D., Brufsky A.M., Hassan S.S., Lundstrom K., Serrano-Aroca Á., Takayama K. (2022). Would new SARS-CoV-2 variants change the war against COVID-19?. Epidemiologia.

[15] Lafuente-Lafuente C., Rainone A., Guérin O., Drunat O., Jeandel C., Hanon O., Belmin J., C-VENH (COVID-19 Vaccine Effectiveness in Older Patients) Investigators (2022). COVID-19 outbreaks in nursing homes despite full vaccination with BNT162b2 of a majority of residents. Gerontology.

[16] Sinha S., Konetzka R.T. (2022). Association of COVID-19 vaccination rates of staff and COVID-19 illness and death among residents and staff in US nursing homes. JAMA Netw. Open.

[17] Mensah A.A., Lacy J., Stowe J., Seghezzo G., Sachdeva R., Simmons R., Bukasa A., O’Boyle S., Andrews N., Ramsay M. (2022). Disease severity during SARS-COV-2 reinfection: A nationwide study. J. Infect..

